# Pectin-based emulsion gels: Formation mechanisms, multiscale characterization, influencing factors and applications

**DOI:** 10.1016/j.fochx.2026.104707

**Published:** 2026-10-27

**Authors:** Tingting Qi, Jingnan Ren, Siyi Pan, Gang Fan

**Affiliations:** Key Laboratory of Environment Correlative Dietology, Ministry of Education, College of Food Science and Technology, Huazhong Agricultural University, Wuhan 430070, China

**Keywords:** Pectin, Emulsion gels, Interfacial interactions, Formation mechanisms, Applications

## Abstract

Emulsion gels combine the kinetic stability of emulsions with the structural integrity of gels by immobilizing oil droplets in a three-dimensional network, thereby limiting conventional emulsion instabilities and supporting delivery of hydrophobic bioactives. Pectin, an abundant and food-approved acidic heteropolysaccharide, is an attractive matrix because of its amphiphilicity and tunable gelation. A critical synthesis focused specifically on pectin-based emulsion gels—covering formation mechanisms, interfacial behaviour, multiscale structure–property relationships and application prospects—is still limited. This review examines preparation, interfacial and gelation mechanisms, multiscale characterization, key influencing factors and translational potential, with emphasis on how pectin molecular architecture (degree of methoxylation (DM), rhamnogalacturonan-I (RG-I) domains and proteinaceous moieties) shapes colloidal behaviour. By identifying current limitations and open questions, it aims to support more rational design of pectin-based emulsion gels for food structuring, nutraceutical delivery and related soft-matter uses.

## 1 Introduction

Emulsion gels are semi-solid or solid colloidal materials formed by converting liquid emulsions into three-dimensional networks through physical, enzymatic, chemical, or hybrid gelation strategies. These composite systems consist of emulsified droplets immobilized within a continuous gelled matrix, thereby integrating the functional advantages of both emulsions and hydrogels. Such architectures provide exceptional structural stability while enabling the simultaneous encapsulation and controlled release of both hydrophobic and hydrophilic bioactive compounds (Kavya et al., 2023; Zhi et al., 2023). By confining oil droplets within a solid-like network, emulsion gels effectively suppress common instabilities such as flocculation, coalescence, Ostwald ripening, and phase separation (Lei et al., 2015). These distinctive properties have positioned emulsion gels as versatile platforms in food, pharmaceutical, and biomedical applications (Hu et al., 2023).

The choice of emulsifier is critical in emulsion gel design. Although synthetic surfactants remain widely used, growing consumer concerns over their safety and long-term health effects have driven increasing interest in natural biopolymers—particularly proteins and polysaccharides—as biocompatible alternatives (Balakrishnan et al., 2017). Among these, pectin, an acidic heteropolysaccharide abundant in plant cell walls, has emerged as one of the most promising candidates (Thakur et al., 1997). Recognized as a safe food additive by the Joint FAO/WHO Expert Committee on Food Additives (1969) with no specified acceptable daily intake limit, pectin exhibits inherent dual functionality as both an emulsifier and a gelling agent.

Structurally, pectin comprises four major domains (Fig. 1): homogalacturonan (HG), a linear α-(1,4)-linked D-galacturonic acid backbone; rhamnogalacturonan I (RG-I), the dominant branched region consisting of rhamnose-galacturonic acid repeating units decorated with neutral sugar side chains (mainly arabinose and galactose); rhamnogalacturonan II (RG-II), a complex branched domain; and xylogalacturonan (XG), a xylose-substituted variant of HG (Jia et al., 2024; Ridley et al., 2001). Its gelling behaviour is primarily governed by the degree of methoxylation (DM). High-methoxy pectin (HMP, DM > 50%) forms gels under acidic conditions (pH 2.0–3.5) and high co-solute concentrations through hydrophobic interactions and hydrogen bonding, whereas low-methoxy pectin (LMP, DM < 50%) undergoes ionic cross-linking via the “egg-box” mechanism with divalent cations such as Ca^2+^ and Mg^2+^ (Chan et al., 2017; Qi et al., 2023). Complementing its gelling capacity, pectin's amphiphilic character—arising from the coexistence of hydrophilic (hydroxyl, carboxyl, amide) and hydrophobic (methyl, acetyl, ferulic acid ester) groups—confers strong emulsifying ability, while its high intrinsic viscosity provides additional steric and electrostatic stabilisation of oil droplets (Ngouémazong et al., 2015).

**Fig. 1:**
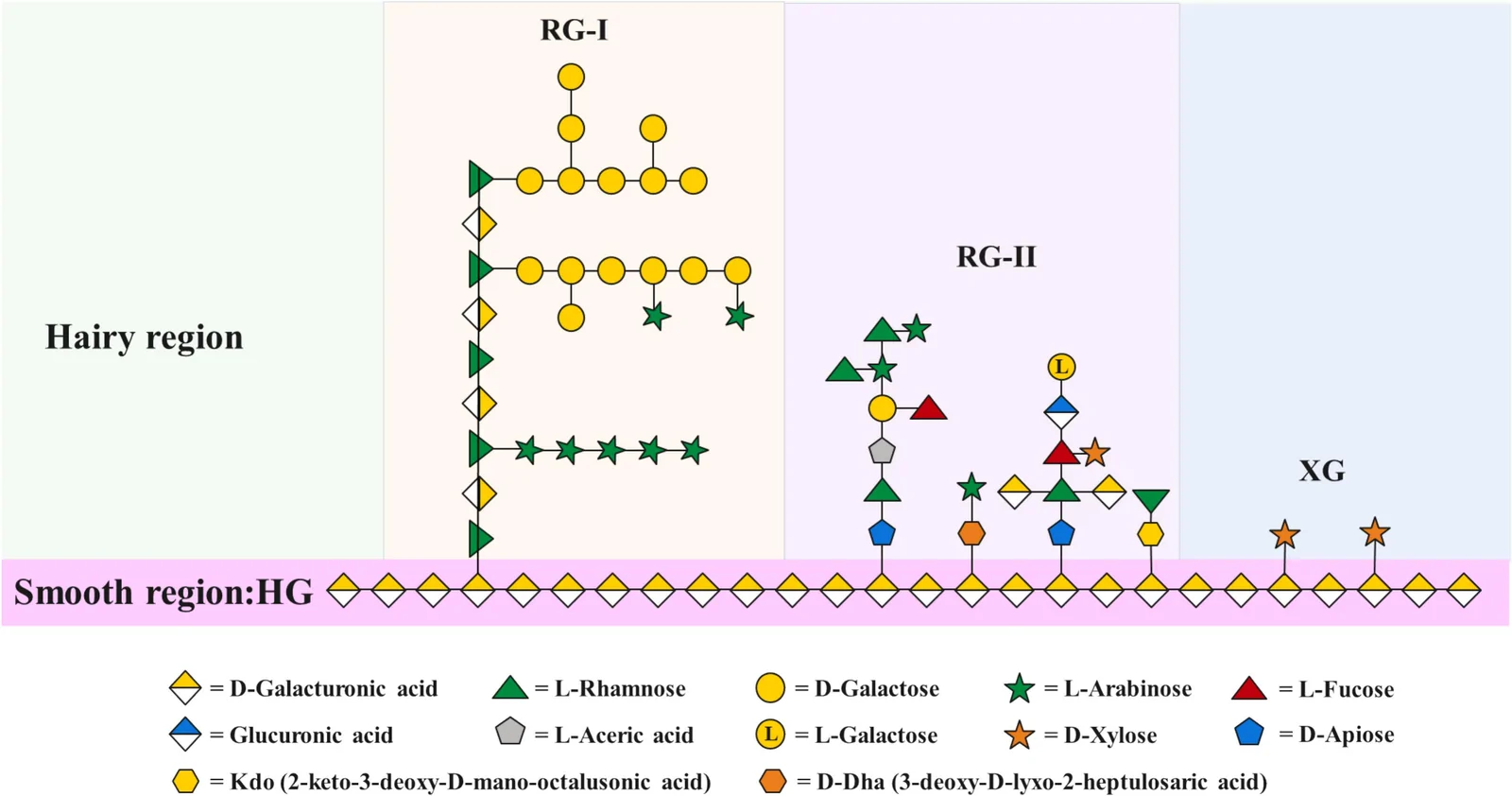
Schematic structure of pectin (Jia et al., 2024) https://doi.org/10.1016/j.foodhyd.2024.110428 Note: HG, homogalacturonan; RG-I, rhamnogalacturonan I; RG-II, rhamnogalacturonan II; XG, xylogalacturonan.

Although many research articles have investigated pectin-based emulsion gels(Qi et al., 2024; Zhang, Zheng, et al., 2022; W. Y. Zhang et al., 2026), and several reviews have covered pectin hydrogels (Ishwarya et al., 2022; Nurul Saadah Said et al., 2023), pectin emulsification (Jia et al., 2024; Ngouemazong et al., 2012; Niu, Hou, et al., 2024), and food-grade emulsion gels (Lin et al., 2020; Lu et al., 2019), systematic attention devoted specifically to pectin-based emulsion gels remains comparatively limited. Relative to previous reviews that focus on pectin hydrogels, pectin emulsification or emulsion gels as a general class, the present work is devoted specifically to pectin-based emulsion gels. It integrates interfacial emulsification with multi-force gelation, treats the oil phase as an active network modulator, and examines unresolved RG-I/HG contributions under realistic oil fractions (Table 1).

**Table 1:** Comparison of recent reviews (2023–2026) with the present work.

**Year / Journal**	**Main scope and strengths**	**Gaps / limitations**	**Difference from the present review**	**References**
2025, *Advances in Colloid and Interface Science*	In-depth focus on Pickering emulsion gels stabilised by protein and polysaccharide-based particles	Narrow scope; does not cover pure-pectin systems, full characterization or broad applications	Covers both pure-pectin and composite systems and systematically compares their interfacial assembly and application scenarios	(Hashemi et al., 2025)
2025, *Comprehensive Reviews in Food Science and Food Safety*	Focused review of protein–pectin delivery carriers for food bioactive ingredients	Narrow scope centred on delivery; limited discussion of pure-pectin emulsion gels and multiscale structure–property coupling	Covers both pure-pectin and composite systems and systematically compares their interfacial assembly and application scenarios	(Wen et al., 2025)
2025, *Food Hydrocolloids*	Transition from hydrogels to emulsion gels; relatively complete structure–mechanism–factors–application framework; covers fat replacement, delivery, 3D printing and preservation coatings	Shallow discussion of interfacial mechanisms; RG-I/HG contradictions in emulsion systems largely unaddressed; oil phase treated as a passive filler	First systematic critique of RG-I/HG contradictions; repositions the oil phase as an **active modulator of network assembly**	(S. X. Huang et al., 2025)
2024, *Food Hydrocolloids*	In-depth multi-scale (macro to molecular) evaluation of pectin emulsifying properties; clear survey of modification strategies and emerging applications	Almost no coverage of gelation processes or overall emulsion-gel performance; lacks network-formation mechanisms	Full chain from emulsification → gelation → multiscale characterization → structure–property coupling	(Jia et al., 2024)
2023, *Food Chemistry*	Broad review of food-grade emulsion gels (preparation, structure, applications); wide coverage	Pectin is only one of many matrices; no pectin-specific in-depth analysis	Pectin-dedicated emulsion-gel review focused on its unique structure and interfacial behaviour	(Zhi et al., 2023)
2023, *Gels*	Systematic discussion of pectin hydrogel gelation mechanisms and food applications	Essentially no emulsion systems or oil-phase effects	Focused on emulsion gels, emphasising the critical roles of the oil phase and interface	(Nurul Saadah Said et al., 2023)

The present review is organised around four themes: (1) preparation and formation mechanisms, including homogenization–interface coupling and multi-force gelation under realistic oil fractions, pH and protein co-solutes; (2) multiscale characterization linking texture, interfacial and bulk rheology, microstructure, and storage stability to process control; (3) structure–environment–property relationships, with emphasis on DM, RG-I architecture and pure versus composite systems; and (4) applications in food structuring, bioactive delivery, coatings and 3D printing, together with translational constraints of evidence quality and cost. The aim is to support more predictive design of pectin-based emulsion gels for food and related soft-matter uses.

## 2 Preparation method and formation mechanism of pectin-based emulsion gels

Pectin-based emulsion gels consist of oil droplets entrapped within a three-dimensional network (Fig. 2). Preparation generally follows two steps: (i) homogenization of a pectin dispersion with an oil phase by high-speed shear homogenization (HSSH), ultrasound homogenization (UH), high-pressure homogenization (HPH) or microfluidization homogenization (MH), and (ii) gelation of the continuous phase by acid, ionic (e.g., Ca^2+^, Mg^2+^, Zn^2+^), or enzymatic. Most studies use emulsion-first then gelation (Lin et al., 2020); pre-forming a hydrogel and then incorporating oil remains rare (X. Zhang et al., 2021). This section reviews emulsification, gelation mechanisms and the distinction between pure pectin and pectin–protein composite systems at the level of interfacial assembly.

**Fig. 2:**
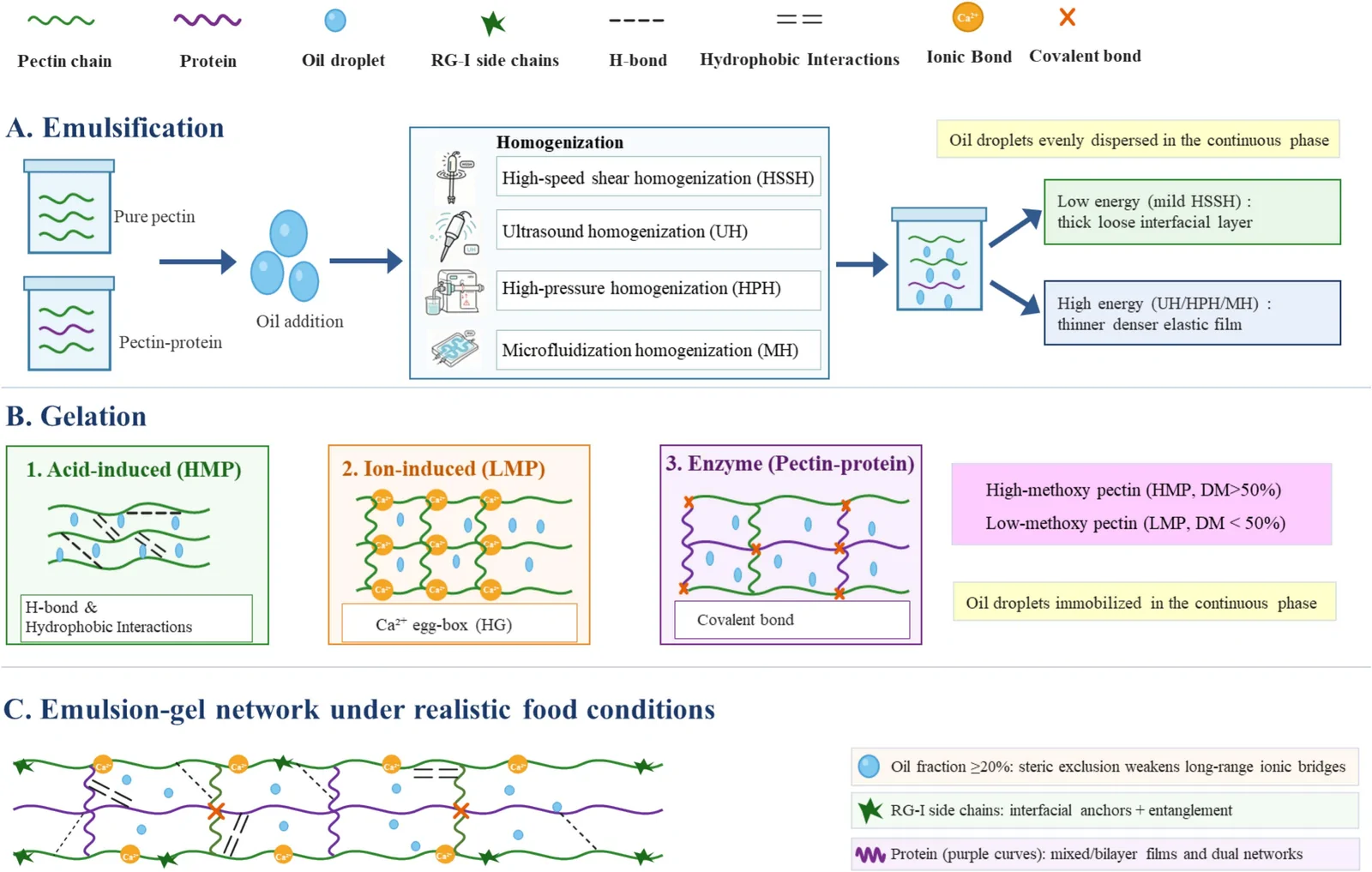
Preparation routes and interfacial/gelation mechanisms of pectin-based emulsion gels.

### 2.1 Preparation and interfacial emulsification mechanisms

Common homogenization methods reduce droplet size, promote pectin adsorption at the oil–water interface and lower interfacial tension through steric and electrostatic stabilisation (Aswathanarayan & Vittal, 2019; Zhi et al., 2023). Energy density also controls pectin degradation and the thickness and density of the adsorbed layer—an often overlooked link between process intensity, interfacial structure and final gel mechanics.

#### 2.1.1 Ultrasound homogenization (UH)

Ultrasound homogenization (UH) is a safe, environmentally friendly, and energy-efficient process operating at acoustic intensities of 10–1000 W cm^−2^ and frequencies of 20–100 kHz, generating cavitation-induced shear and microstreaming (Geng et al., 2022; Higuera-Barraza et al., 2016). High-intensity UH (150–600 W, 20 kHz, pulsed 4 s on/2 s off) applied to soybean protein isolate–citrus pectin emulsions not only fragments large droplets into smaller, more uniform particles but also unfolds pectin chains, reducing interfacial tension and strengthening droplet–matrix interactions via hydrogen bonding, hydrophobic forces, and van der Waals attractions. Subsequent thermal gelation yields finer networks with improved mechanical properties (Zhang et al., 2021). Recent work further confirms that ultrasonic-assisted treatment of *Haematococcus pluvialis* protein–pectin complexes produces highly stable Pickering emulsions with excellent interfacial activity and 3D-printing suitability (B. Li et al., 2025). Ultrasound-assisted pectin-containing DHA nanoemulsions can improve oxidative stability under storage and in vitro digestion conditions (SirajdeenAsmath & Radhakrishnan, 2026). Potato pectin at 40 W/L for 20 min gave a favourable balance of fragmentation and physical stability (Zhang, Yang, et al., 2025).

#### 2.1.2 High-speed shear homogenization (HSSH)

High-speed shear homogenization (HSSH) offers operational simplicity and speed but frequently requires coupling with secondary methods to achieve long-term stability (Córdova et al., 2020; Ma et al., 2023). Mild HSSH (typically 8000–12,000 rpm for short times) generally causes limited depolymerisation, with Mw reductions often remaining below about 15–20%, and yields relatively thick, loosely packed interfacial layers (J. Chen et al., 2014). For instance, stable W₁/O/W₂ double emulsions were prepared using HSSH (12,000 rpm, 2 min) followed by HPH (150 MPa) with *Nicandra physalodes* seed pectin and walnut oil; the resulting emulsions exhibited no visible phase separation after 28 days at 4 °C and were successfully converted into 3D-printable gels upon glucono-δ-lactone (GDL) addition(J. Li et al., 2023). This example illustrates that HSSH is best understood as a coarse emulsification step that often benefits from subsequent refinement, rather than as a stand-alone strategy for obtaining highly stable systems.

#### 2.1.3 High-pressure homogenization (HPH)

High-pressure homogenization (HPH) exploits intense shear, cavitation, and turbulence to further refine droplets. It is routinely combined with HSSH: coarse emulsions are first formed by shear and then passed through a microfluidizer (e.g., 100 MPa, three cycles) to produce fine, mechanically robust emulsions (L. Feng et al., 2019; Ren et al., 2022). High-intensity UH and multi-pass HPH (e.g. 50–150 MPa) can markedly reduce Mw by 30–60% and trimming neutral-sugar side chains, thereby generating thinner, denser interfacial films (Wang, Feng, et al., 2021; Yan et al., 2021). For example, High-methoxy pectin W₁/O/W₂ emulsions were obtained by pre-shearing at 8000 rpm followed by HPH (35 MPa, two passes), and remained homogeneous with high internal droplet loading after one month of storage at 4 °C (Massel et al., 2021). Recent studies combining UH and HPH have shown that these sequential treatments significantly enhance the structural compactness and long-term stability of pectin-based emulsion-templated systems (Abou-Elsoud et al., 2025).

#### 2.1.4 Microfluidization homogenization (MH)

Microfluidization homogenization (MH) (also termed dynamic high-pressure microfluidization) forces the coarse emulsion through interaction chambers with micro-channels under ultra-high pressures (typically 50–200 MPa), generating intense shear, cavitation, impact and instantaneous pressure drop that further reduce droplet size and narrow the size distribution. When applied to pectin systems, microfluidization not only refines oil droplets but can also partially depolymerize pectin chains, disrupt aggregates and increase interfacial loading. Combined MH and moderate ultrasonication of citrus pectin reduced Mw (e.g. from ∼485 kDa to ∼238 kDa) and increased interfacial loading (Wang, Feng, et al., 2021). Microfluidization has also been shown to modify pectin–protein complexes (e.g., pectin–lactoferrin), enhancing dispersibility and interfacial activity depending on the treatment sequence. In addition, microfluidization is occasionally used to generate fine nanoemulsions that are subsequently thickened or gelled with pectin, yielding systems with improved long-term physical stability (Sanchez et al., 2022). Dedicated studies that use MH as the primary emulsification step for pectin-stabilised emulsion gels remain fewer than those on HSSH, UH and HPH. Most available work focuses on using microfluidization to pre-modify pectin itself rather than to emulsify the oil phase in the presence of pectin. Consequently, systematic data linking microfluidization energy density to interfacial film thickness, dilatational rheology and subsequent gelation behaviour are still scarce and represent a clear opportunity for future research.

#### 2.1.5 Process energy and interfacial architecture

Overall, homogenization does more than reduce droplet size: it also reshapes pectin chains and the interfacial film. Low-energy HSSH generally limits depolymerisation and favors thicker, more sterically stabilising layers, whereas high-energy UH, HPH and MH can cut molecular weight and neutral-sugar side chains and promote thinner, denser, more elastic films. The useful process window is therefore the range in which droplet size is refined without excessive loss of interfacial steric bulk. Most studies still report only final droplet size or bulk gel modulus, without parallel data on Mw, side-chain composition and interfacial rheology under the same conditions. Establishing such quantitative structure–process–interface relationships is therefore essential if homogenization is to be transformed from an empirical size-reduction step into a deliberate tool for engineering interfacial architecture.

### 2.2 Gelation mechanisms

Pectin functions as a versatile natural gelling agent, yet the mechanisms that convert a pectin-stabilised emulsion into a self-supporting emulsion gel are far more complex than the classical acid-, ion- or enzyme-induced categorisations suggest. In oil-free hydrogels these routes can be discussed in relative isolation. In emulsion gels the same molecular forces operate concurrently, compete with one another, and are continuously re-weighted by pH, ionic strength, oil-volume fraction and protein co-solutes. Understanding gelation under realistic food conditions therefore requires an analysis of competing interactions rather than sequential, single-force descriptions.

#### 2.2.1 Classical driving forces and their conventional regimes

High-methoxy pectin (HMP, DM > 50%) traditionally gels under acidic conditions (pH ≤ 3.5) in the presence of high co-solute concentrations (typically≥55–65% sugar). Low pH protonates the majority of carboxyl groups, diminishing electrostatic repulsion, while high sugar lowers water activity and promotes chain association. Hydrophobic interactions between methyl ester groups dominate above approximately 50 °C and are subsequently reinforced by hydrogen bonding upon cooling, yielding the characteristic thermo-reversible HMP network (Einhorn-Stoll, 2018; Oakenfull & Scott, 1984; Sperber et al., 2009). This sequence is also termed cold-set gelation (Kastner et al., 2014). Ethanol/acid mixtures can likewise drive HMP gelation, although hydrogen bonding then becomes the predominant stabilising force (W. X. Jiang et al., 2021).

Low-methoxy pectin (LMP, DM < 50%) generally gels through ionic bridging with divalent cations such as Ca^2+^, Mg^2+^, or Zn^2+^ according to the classic “egg-box” model, in which one cation coordinates carboxyl groups from adjacent galacturonic acid chains. The classic egg-box model accounts for the strong dependence of gel strength on both the absolute number of non-methoxylated residues and their block-wise distribution (Cao et al., 2020). Moderate calcium concentrations produce homogeneous, elastic networks, whereas excess Ca^2+^ can induce local over-cross-linking and micro-syneresis. Certain LMPs can nevertheless form weak gels at pH < 3.5 even in the absence of added calcium, relying primarily on hydrogen bonding and hydrophobic interactions (Vriesmann & Petkowicz, 2017).

Enzyme-mediated routes provide a third classical pathway. Laccase or peroxidase catalyses the oxidative coupling of feruloyl residues present on RG-I side chains, generating covalent diferulic bridges that permanently stabilise the network. Alternatively, pectin methylesterase (PME) can be used to generate additional calcium-sensitive blocks in situ, thereby converting an HMP into a more ionically reactive LMP without the need for chemical de-esterification. These covalent or enzymatically tuned junctions are largely irreversible and confer enhanced thermal and mechanical robustness (H. Chen et al., 2019; L. Feng et al., 2019).

These textbook regimes remain useful reference points. They were developed almost entirely for oil-free hydrogels and therefore map only part of the force balance inside an emulsion gel.

#### 2.2.2 Interference by the oil phase and protein co-components

Two food-matrix factors in particular break single-force descriptions when hydrogel mechanisms are applied to emulsion gels.

The dispersed oil phase is not a passive filler. Above an oil volume fraction of about 20%, steric exclusion by the closely packed droplets shortens the effective contour length available for inter-chain junctions. Long-range ionic bridges are preferentially disrupted, whereas shorter-range hydrogen bonds and hydrophobic contacts remain relatively intact. In addition, droplet–matrix interactions can template local chain orientation, generating anisotropic network domains that classical bulk-gel models cannot predict. Recent interfacial-rheology and cryo-microscopy studies confirm that the oil–water interface itself becomes an active participant in network assembly rather than a mere boundary (Feng et al., 2024; Huang et al., 2025). The same pectin that forms a homogeneous hydrogel without oil may therefore give a weaker or more heterogeneous emulsion gel once a substantial oil phase is present.

Protein co-solutes, almost always present in real formulations, compete for interfacial area and add electrostatic, hydrophobic or covalent linkages. Near the protein isoelectric point, protein–pectin electrostatic complexes form rapidly at the oil–water interface, often dominating early adsorption and subsequently serving as nucleation sites for continuous-phase network growth. Sequential or simultaneous adsorption of whey, soy or potato proteins with low-methoxy pectin elevates both dilatational and shear interfacial moduli, producing mixed or bilayer films whose mechanical strength exceeds that of either component alone (Li & Van der Meeren, 2022; Eichhorn et al., 2025). Laccase- or transglutaminase-mediated covalent cross-linking between feruloyl residues on pectin and tyrosine residues on proteins can further generate dual-network architectures whose storage modulus, fracture stress and water-holding capacity substantially surpass those of single-component gels (Chen et al., 2019; Feng et al., 2021; Zhang, Zhao, et al., 2025). Excess unbound protein, however, may strip pectin from the interface or cause bridging flocculation before gelation locks the structure. Protein–pectin interactions thus often re-rank the classical driving forces and can control both interfacial integrity and bulk performance in multi-component systems.

#### 2.2.3 Competitive and synergistic interactions under realistic food pH and ionic strength

In the pH range of most foods (about 3.5–6.5) carboxyl groups are only partially protonated. Consequently, residual electrostatic repulsion coexists with emerging hydrophobic contacts and hydrogen bonds, so that acid- and ion-induced mechanisms operate together rather than in sequence (Cao et al., 2020; Capel et al., 2006). The relative weight of each contribution is further modulated by ionic strength. At low salt concentrations, chains remain extended, favouring intermolecular hydrogen bonding; moderate salt screens charges and can promote hydrophobic association; above a critical threshold, chain collapse may weaken long-range network connectivity (Garnier et al., 1993). Mixed acid/ion experiments show non-additive behaviour. Moderate Ca^2+^ concentrations can reinforce an acid-set network by bridging residual charged domains, yet excess calcium at low pH frequently produces local over-crosslinking and micro-phase separation, reducing macroscopic homogeneity and water-holding capacity (Capel et al., 2006; Liu, Lei, et al., 2025). Temperature history adds another layer of complexity: hydrophobic interactions are strengthened by heating, while hydrogen bonds are favoured by cooling, so that the final network topology reflects the entire thermal trajectory rather than a single equilibrium state (Cao et al., 2020). Thus, the dominant stabilising force is set by the combination of pH, free divalent cations, ionic strength and temperature—not by DM alone.

#### 2.2.4 Unresolved contradictions surrounding RG-I versus HG contributions

A particularly instructive yet still unresolved tension exists between the classical HG-centric egg-box model and the emerging evidence for RG-I side-chain contributions. In oil-free LMP hydrogels, Ca^2+^-mediated junctions form primarily within non-methoxylated HG domains, while RG-I regions were long regarded as steric obstacles. However, studies on citrus RG-I-enriched pectin (DM 13–15%) have shown that the same material can gel under both acid- and ion-induced conditions. Ion-induced networks are stabilised by egg-box junctions together with entanglement of RG-I side chains. Acid-induced gels, by contrast, rely mainly on hydrophobic interactions and hydrogen bonding within HG regions, which are further tightened by side-chain collapse at low pH (J. Q. Zheng et al., 2020).

These dual mechanisms do not transfer unchanged to emulsion gels. Under the high oil fractions discussed above, DM-centric egg-box predictions become unreliable, because continuous ionic bridging in the aqueous phase is readily interrupted (L. P. Feng et al., 2024; S. X. Huang et al., 2025). Simultaneously, RG-I neutral sugar side chains can act as interfacial anchors that enhance chain entanglement and water binding even at reduced Ca^2+^ levels (Qi et al., 2024). RG-I branching may therefore hinder classical egg-box formation in pure hydrogels yet become advantageous once an oil phase is present. These observations are still rarely compared under matched oil fraction, pH and ionic strength, so a quantitative ranking of HG bridging, RG-I entanglement and interfacial anchoring remains lacking. Key contradictions between HG-centred and RG-I-inclusive views are summarised in Table 2.

**Table 2:** Unresolved contradictions surrounding RG-I versus HG contributions.

**Issue**	**Classical view (HG-centred)**	**RG-I-related evidence**	**Specificity in emulsion gels**	**Open questions**
Acid-induced gelation	Driven mainly by hydrophobic contacts and hydrogen bonding within HG	At low pH, RG-I side-chain collapse tightens intermolecular contacts and supports network formation	Interfacially adsorbed RG-I chains can act simultaneously as anchors and steric stabilisers when an oil phase is present	Direct in-situ evidence that acid- and ion-induced forces cooperate inside emulsion gels remains limited
Ion-induced gelation	Egg-box junctions form only in non-methoxylated HG segments	RG-I side chains reinforce the network by entanglement and hydrogen bonding; stable gels can form even at reduced Ca^2+^ levels	At oil volume fractions ≳ 20%, droplet steric exclusion disrupts continuous egg-box bridging in the aqueous phase	Side-chain length/branching versus Ca^2+^ dose–response curves under matched oil fractions are still lacking
Role of neutral-sugar side chains	Often treated as steric obstacles that hinder gelation	Moderately long galactan/arabinan chains enhance entanglement and water holding	In emulsion gels, the same side chains affect both interfacial-layer thickness and continuous-phase network density	How optimal side-chain length/branching varies with oil type and volume fraction is not established
Protein / feruloyl residues	Secondary structural details	Provide sites for covalent cross-linking in composite systems	In high-oil or Pickering systems, protein–pectin interfacial complexes often dominate stabilisation	Relative weights of covalent versus non-covalent contributions under realistic ionic strength are not quantified

*Note.* Compiled from Cao et al. (2020); Capel et al. (2006); Chen et al. (2019); Eichhorn et al. (2025); Einhorn-Stoll (2018); Feng, Jia, Yan, Yan and Yin, 2021, Feng et al., 2024; Funami et al. (2007); Hashemi et al. (2025); Huang et al. (2025); Li and Van der Meeren (2022); Ngouémazong et al. (2015); Oakenfull and Scott (1984); Qi et al. (2024); Zhang, Zhao, et al. (2025); Zheng et al. (2020). Absolute thresholds remain formulation-dependent.

#### 2.2.5 Toward a multi-force, multi-component framework

Overall, gelation of pectin-based emulsion gels in food systems is multi-force and multi-component. Hydrogen bonding, hydrophobic association, ionic bridging and covalent cross-linking operate concurrently; their balance is continuously shifted by local pH, ionic strength, oil-volume fraction and protein identity; and the fine structure of both HG and RG-I domains further modulates each contribution. Predictive design needs to move beyond DM-centric or single-inducer models toward multiscale frameworks that include interfacial rheology, droplet–matrix interactions and competitive adsorption of co-solutes. Only such an integrated perspective can rationalise the diverse mechanical and release behaviours reported for pectin emulsion gels and guide their reliable translation into real food matrices.

### 2.3 Pure pectin and pectin–protein composite systems: Interfacial assembly and network formation

Pure pectin and pectin–protein composites follow different interfacial assembly routes and build continuous-phase networks by different dominant forces, even though both are often prepared by the same emulsification–gelation sequence.

In pure pectin systems, interfacial adsorption is driven primarily by the intrinsic amphiphilicity of the polysaccharide—hydrophobic methyl, acetyl and feruloyl groups, together with residual proteinaceous moieties, anchor the chain at the oil–water interface, while the hydrophilic backbone and RG-I side chains extend into the aqueous phase to provide steric and electrostatic stabilisation. Subsequent gelation relies on acid-induced hydrophobic interactions and hydrogen bonding (HMP) or Ca^2+^-mediated egg-box junctions (LMP), further modulated by RG-I entanglement. The resulting networks are relatively homogeneous but often show limited mechanical robustness under thermal, freeze–thaw or high-ionic-strength conditions, and long-term stability is highly sensitive to oil-volume fraction.

In pectin–protein composite systems the sequence is inverted and more complex. Proteins (soy protein isolate, whey protein, pea protein, zein, etc.) typically adsorb first and rapidly lower interfacial tension. Pectin then interacts with the pre-adsorbed protein layer through electrostatic complexation (especially near the protein isoelectric point), hydrogen bonding, hydrophobic association or, in the presence of laccase or transglutaminase, covalent cross-linking between feruloyl residues and tyrosine. The outcome is a mixed or multilayered interfacial film whose dilatational elasticity and resistance to displacement far exceed those of either component alone (Eichhorn et al., 2025a; Y. Li et al., 2022). In the continuous phase, protein–pectin coacervates or covalent dual networks form, producing denser microstructures with superior water-holding capacity, thermal stability and tolerance to processing stresses (Hashemi et al., 2025; Zhang, Yang, et al., 2025).

Pectin–protein composite emulsion gels form in three overlapping stages. First, protein and pectin compete for or sequentially occupy the oil–water interface; the outcome depends on the protein-to-pectin ratio, the order of addition and the pH. Second, unadsorbed protein and pectin strengthen the continuous phase by forming physical complexes or, when enzymes such as laccase or transglutaminase are used, covalent dual networks. Third, under shear, heat or freeze–thaw stress, the mixed interfacial film helps prevent coalescence, while the dual network absorbs energy through weak physical bonds and stronger covalent links. How much stability comes from electrostatic binding versus covalent cross-linking, which protein–pectin ratio gives the strongest interface, and how leftover unbound protein affects the continuous phase are still not well quantified, so fully predictive design of composite emulsion gels remains difficult.

## 3 Physicochemical properties and multiscale characterization

Comprehensive characterization of pectin-based emulsion gels is essential for elucidating their formation mechanisms, structure–property relationships, and performance in processing and applications. Multiscale techniques span macroscopic sensory and textural assessments to microscopic rheological, microstructural, and stability analyses, collectively bridging interfacial phenomena with bulk functionality.

### 3.1 Sensory evaluation

Visual and manual sensory assessment remains the most accessible initial screening tool, providing rapid insights into color, transparency, morphology (lump, granule, or fluid-like), and macroscopic stability (phase separation, creaming, or aggregation). Approximate viscoelasticity can also be gauged by hand. Nevertheless, sensory evaluation is inherently subjective and qualitative; quantitative, instrument-based methods are indispensable for reproducible and accurate characterization (Yiu et al., 2023).

### 3.2 Texture

Texture is a critical consumer-relevant attribute and a key performance indicator for emulsion gels. Texture profile analysis (TPA) via compression testing is the standard approach, quantifying parameters such as hardness (gel strength), springiness (elasticity), fracturability, adhesiveness, chewiness, cohesiveness, gumminess, and resilience. Hardness and springiness are the most frequently reported indices. Baydin et al. (2022) conducted a comprehensive texture analysis of pectin-based emulsion gels using TPA, providing valuable fundamental insights into their potential applications in both food and pharmaceutical fields. N. S. Said and Lee (2025) developed pea protein isolate–high-methoxy pectin conjugates via the Maillard reaction and employed TPA to demonstrate that the resulting soft food gels exhibited significantly improved cohesiveness and reduced hardness, making them suitable for dysphagia-friendly textures. Similarly, Y. Chen et al. (2025) used TPA to evaluate plant-based whipped creams formulated with pea protein–high-methoxy pectin emulsion gels, showing that appropriate oil phase volume fractions yielded hardness and adhesiveness comparable to traditional dairy-based products.

### 3.3 Rheology

#### 3.3.1 Bulk rheology

Rheological analysis is the most widely used technique for characterizing the viscoelastic behaviour of pectin-based emulsion gels under processing and storage conditions. Measurements typically begin with strain sweeps to determine the linear viscoelastic region (LVR), followed by small-amplitude oscillatory shear (SAOS) tests within the LVR to quantify the storage modulus (G′), which reflects the elastic, solid-like response, and the loss modulus (G″), which represents the viscous, liquid-like response. The damping factor (tan δ = G″/G′) provides a sensitive indicator of the gel-to-sol transition, with the crossover point (tan δ = 1) conventionally regarded as the gel point (Holst et al., 2006). Additional rheological tests, including large-amplitude oscillatory shear (LAOS), frequency sweeps, time sweeps, and creep-recovery experiments, offer deeper insights into shear sensitivity, structural evolution, and recovery capacity of the gel network.

Low-methoxy pectin, with its higher charge density, was demonstrated to significantly enhance gel strength in whey protein-stabilised bilayer emulsion gels compared with high-methoxy variants (Kadiya & Ghosh, 2022). Rice bran protein–pectin conjugated microgel particles were reported to produce emulsion gels with superior rheological properties, including markedly elevated G′ and excellent thermal stability up to 140.69 °C, underscoring the critical role of protein–pectin interfacial interactions in reinforcing the gel network (Han et al., 2026). Similarly, Ca^2+^-induced citrus pectin emulsion gels were systematically investigated, revealing that both RG-I side chain content and Ca^2+^ concentration synergistically modulate G′, microstructural homogeneity, and limonin release kinetics, thereby establishing quantitative structure–rheology–delivery relationships (Zhang et al., 2026). Recent studies underscore that rheological parameters directly reflect interfacial network density and droplet–matrix interactions, yet most work still emphasizes bulk rather than interfacial rheology (Feng et al., 2024; Kavya et al., 2023).

#### 3.3.2 Interfacial rheology

While bulk rheology characterises the macroscopic mechanical response of the continuous-phase network, interfacial rheology quantifies the viscoelastic properties of the adsorbed film that ultimately governs droplet coalescence, film drainage and long-term emulsion stability. Two complementary approaches are commonly employed: dilatational rheology, which probes the response of the interface to expansion and compression, and shear interfacial rheology, which measures in-plane resistance to deformation (El Omari et al., 2022; Sagis & Fischer, 2014).

Dilatational measurements (typically performed with a pendant-drop or Langmuir-trough apparatus) yield the elastic (E'or E_d_') and viscous (E″) moduli of the adsorbed layer. A high dilatational elastic modulus indicates a cohesive, solid-like film capable of withstanding the local deformations that occur during droplet collision. For instance, dilatational rheology of commercial citrus pectins has shown that LMP develop higher interfacial elasticity than HMP under acidic conditions, reflecting stronger intermolecular associations at the interface (Baldino et al., 2018). In protein–pectin systems, sequential adsorption of whey protein followed by low-methoxy pectin markedly elevates the dilatational viscoelastic moduli, with maximum values obtained near the protein isoelectric point (H. Li & Van der Meeren, 2022). Complementary shear interfacial rheology further reveals that potato-protein–pectin conjugates form films that adsorb faster than protein alone and exhibit distinct plastic responses under large deformation, the compactness of the secondary pectin layer being strongly pH- and pectin-type-dependent (Eichhorn et al., 2025b). Recent high-internal-phase emulsion studies have additionally demonstrated quantitative correlations between both dilatational (E_d_') and shear interfacial (G_i_') moduli and the bulk storage modulus (G′) of the final gel, underscoring the predictive value of interfacial rheology for macroscopic mechanical performance.

Despite its central importance, interfacial rheology remains under-utilised in the pectin emulsion-gel literature. Most studies still rely on endpoint droplet size or bulk G′ without reporting the intermediate interfacial mechanical properties. Systematic dilatational and shear measurements under realistic pH, ionic strength and protein-to-pectin ratios are therefore essential if structure–function relationships are to move from qualitative observation to predictive design.

### 3.4 Microstructure

Microstructural characterization reveals droplet size, shape, distribution, and interfacial interactions, which profoundly influence flavour release, processability, appearance, stability, and overall functionality. Particle size distribution is commonly measured using laser diffraction (suitable for micrometer-range particles) and dynamic light scattering (effective for nanometer and sub-micrometer particles), the latter yielding the *Z*-average diameter and polydispersity index (PDI). Complementary imaging techniques then visualise the three-dimensional organisation of the system at multiple length scales. Optical microscopy offers a rapid first assessment of bead or droplet size under ambient conditions. Conventional scanning electron microscopy (SEM) reveals network-wall thickness, pore size and overall porosity, yet the necessary drying or chemical fixation steps can introduce artefacts that distort the native hydrated architecture. Cryogenic scanning electron microscopy (Cryo-SEM) further eliminates drying artefacts: the sample is rapidly frozen and the fracture surface is imaged in the vitreous state, preserving the true spatial arrangement of droplets, interfacial films and continuous-phase junctions. Confocal laser scanning microscopy (CLSM) overcomes the lack of chemical specificity by employing selective fluorophore labelling, thereby allowing spatial discrimination of pectin, protein and lipid phases within the same field of view.

These complementary tools have been applied to a range of pectin emulsion-gel systems. Using laser diffraction and optical microscopy, Hou et al. (2016) showed that high-pressure homogenization (two passes at 300 bar) produces significantly smaller oil droplets than high-speed shear alone, thereby increasing interfacial area, strengthening droplet–matrix binding and generating a more compact gel network that effectively retards aroma diffusion. SEM further revealed that higher emulsion concentrations yield thicker network walls and smaller pores (Pan et al., 2021). Cryo-SEM has been employed to visualise ordered nanoparticle coatings and inter-droplet bridging networks in zein–hawthorn-pectin systems (Y. Jiang et al., 2020). CLSM has likewise enabled the direct visualisation of laccase-induced sugar-beet-pectin networks that effectively entrap and protect oil droplets (M. Zhang, Yin, et al., 2022).

Collectively, laser diffraction, dynamic light scattering, optical microscopy, SEM, Cryo-SEM and CLSM provide a multi-scale morphological framework that links pectin molecular architecture and processing history to the compactness, homogeneity and long-term stability of the final emulsion gel.

### 3.5 Stability

Stability under thermal, mechanical, gastrointestinal, water-related and long-term storage stresses collectively determines the practical utility of pectin-based emulsion gels. Resistance to heat and shear governs processability, gastrointestinal integrity controls delivery performance, water-holding capacity and freeze–thaw behaviour affect texture, while long-term storage stability—resistance to creaming, coalescence, oiling-off, syneresis and oxidative deterioration over weeks to months—ultimately decides whether the emulsion gels can survive the time–temperature history of real food.

#### 3.5.1 Thermal stability

Thermal stability is commonly evaluated using thermogravimetric analysis (monitoring mass loss with temperature) or differential scanning calorimetry (DSC), which quantifies transitions such as melting, decomposition, or cross-linking. It was shown that high-intensity ultrasound treatment raised the denaturation temperature of pectin–soybean protein isolate emulsion gels to 131.9 °C, indicating improved thermal resistance (Zhang et al., 2021).

#### 3.5.2 Mechanical stability

Mechanical stability refers to the gel's resistance to external forces such as shear, compression, or centrifugal stress without significant structural breakdown or phase separation. It is typically assessed by rheological tests (large-amplitude oscillatory shear or creep-recovery) and centrifugation assays. It was demonstrated that double cross-linking with calcium and transglutaminase significantly improved mechanical strength and apparent viscosity in citrus pectin–zein emulsion gels (Kuang et al., 2024).

#### 3.5.3 Water-state and hydration stability

Water distribution, mobility, water-holding capacity and swelling behaviour collectively govern texture, syneresis, freeze–thaw resistance and overall network integrity of pectin-based emulsion gels. Low-field nuclear magnetic resonance (LF-NMR) relaxometry quantifies water states non-destructively through transverse relaxation times (T₂). Shorter T₂₁ and T₂₂ components correspond to bound and immobilized water within the pectin network, while longer T₂₃/T₂₄ populations reflect free water. An increase in oil fraction or cross-link density systematically shifts the T₂ spectrum toward shorter times, indicating stronger water binding and higher structural stability (Qi et al., 2024). Complementary centrifugation assays measure macroscopic water-holding capacity; lower mass loss after centrifugation reflects stronger retention. Increasing oil content from 5% to 25% has been reported to raise water-holding capacity from approximately 75% to 95%. Swelling kinetics further report network porosity and controlled-release potential: denser, highly cross-linked structures exhibit lower swelling rates and more sustained release profiles (Zhang, Yin, et al., 2022). Together, LF-NMR, water-holding capacity and swelling analysis provide a quantitative bridge between molecular architecture, network density and macroscopic stability that cannot be obtained by any single method alone.

#### 3.5.4 Gastrointestinal stability

Gastrointestinal stability and digestion behaviour are particularly important for bioactive delivery. In vitro simulated digestion models (oral, gastric, and intestinal phases) are widely used to evaluate structural integrity, lipid hydrolysis, bioactive release, and bioaccessibility. RG-I-enriched citrus pectin emulsion gels were demonstrated to maintain good stability under centrifugal, pH, and thermal stress while providing controlled release of nobiletin during simulated gastrointestinal digestion (Qi et al., 2024). Pectin-based emulsions were further revealed to undergo progressive destabilization in the upper gastrointestinal tract due to reductions in pectin molecular weight and degree of esterification, which decrease interfacial adsorption and promote network restructuring with Ca^2+^ in the small intestine (Du et al., 2025).

#### 3.5.5 Storage stability

Storage stability refers to the resistance of emulsion gels to creaming, coalescence, oiling-off, syneresis, network weakening and oxidative deterioration over weeks to months at refrigeration (4 °C) or ambient temperature. Practical indicators include changes in droplet-size distribution, creaming index, visual phase separation, and, for oil-rich systems, peroxide value or TBARS. In pure pectin systems, storage performance is highly sensitive to oil-volume fraction, pectin concentration and network continuity. Many moderate-oil formulations remain homogeneous for 28 days at 4 °C, yet higher oil loads or incomplete interfacial coverage frequently lead to progressive droplet coalescence and serum separation. RG-I-enriched or higher-molecular-weight pectins generally improve long-term stability by enhancing steric hindrance and continuous-phase elasticity. Pectin–protein composite emulsion gels typically outperform single-component systems. The mixed interfacial film and dual-network architecture markedly retard coalescence and syneresis; fish-gelatin–pectin emulsions, for example, remained fluid and free of phase separation throughout extended storage at 25 °C when pectin was present, whereas gelatin-only controls stratified and underwent liquid-to-gel transition within 22–28 days (S. Huang et al., 2022).

#### 3.5.6 Integrated stability assessment

Collectively, thermal, mechanical, water-related, gastrointestinal and storage stability assessments provide a comprehensive picture of how pectin-based emulsion gels respond to the full spectrum of stresses encountered during processing, digestion and shelf life. No single method is sufficient; only the integrated use of DSC, large-amplitude rheology, LF-NMR, simulated digestion models and multi-week storage trials can link molecular architecture and interfacial design to macroscopic performance. Future work should therefore prioritise standardised, multi-technique stability protocols that simultaneously track structural integrity, water-state evolution and oxidative markers under realistic time–temperature histories.

### 3.6 Molecular simulation approaches

Experimental techniques alone cannot resolve the atomic-scale orientation of HG/RG-I domains at the oil–water interface, nor the competitive binding of protein and pectin segments. All-atom and coarse-grained molecular dynamics (MD) simulations are beginning to fill this gap.

Recent simulations of related polysaccharide systems have mapped the preferential adsorption of hydrophobic moieties onto the oil surface, the extension of hydrophilic groups into the aqueous phase, and the energy barriers that oppose droplet coalescence. All-atom MD of cellulose-coated oil droplets showed that amphiphilic chain orientation—hydrophobic faces contacting oil and hydroxyl groups contacting water—stabilises the interfacial film (Miyamoto et al., 2017). Coarse-grained MD of hydrophobically modified sodium alginate emulsions further demonstrated that denser interfacial coverage raises the coalescence energy barrier and creates a cushioning effect at the collision interface. Conformational MD of homogalacturonan likewise revealed a shift from a 2-fold helical structure under dry conditions to a 3-fold helix in water, driven by hydrogen-bond formation with solvent (F. He et al., 2025).

Although direct molecular-dynamics studies of intact pectin emulsion gels remain scarce, the transferable insights already obtained from related polysaccharide systems point to several clear research priorities. Future simulations should move beyond single-component or simplified interfaces and explicitly incorporate realistic oil-volume fractions, mixed HG/RG-I architectures, residual protein moieties and physiologically relevant ionic conditions. Multi-scale approaches that couple all-atom descriptions of the interfacial film with coarse-grained representations of the continuous-phase network would allow the simultaneous capture of local chain orientation, side-chain entanglement and long-range droplet–matrix interactions. Equally important is the systematic mapping of free-energy barriers to coalescence and film rupture as functions of degree of methoxylation, RG-I branching density and calcium concentration. When such trajectories are quantitatively linked to experimental dilatational moduli, Cryo-SEM morphology and bulk gel strength, molecular simulation will evolve from a retrospective illustration into a predictive design tool capable of guiding the rational engineering of pectin-based emulsion gels for food, delivery and 3D-printing applications.

### 3.7 Critical appraisal

Although the literature has accumulated extensive descriptive data, a major weakness lies in the widespread use of single-parameter optimization strategies—often limited to reporting increases in G′, reductions in droplet size, or improved water-holding capacity—without systematically linking these outcomes to underlying pectin molecular architecture or network assembly pathways. Compounding the issue, wide variation in experimental conditions (temperature, frequency, strain amplitude, pretreatment, and imaging parameters) renders meaningful cross-study comparisons extremely difficult. From a review perspective, the field urgently requires a conceptual shift: characterization must evolve from a mere checklist of techniques into a coherent mechanistic framework that explicitly explains how pectin chemistry dictates interfacial assembly, network formation, and long-term functional stability.

## 4 Influencing factors

The properties of pectin-based emulsion gels are governed jointly by pectin intrinsic structure and external formulation and processing conditions (Fig. 3). A clear separation of these two classes of factors is useful for rational design, but they do not act independently: environmental variables continuously re-weight the contribution of each structural motif.

**Fig. 3:**
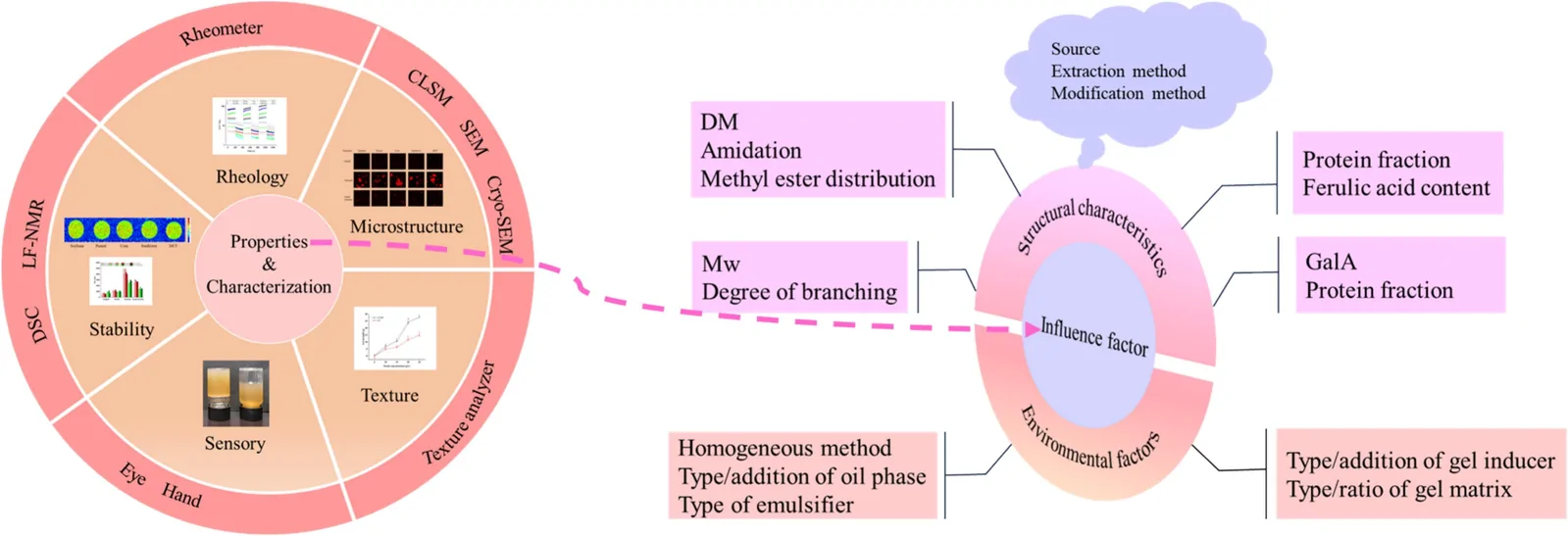
Factors affecting the properties of pectin-based emulsion gels.

### 4.1 Intrinsic structural characteristics of pectin

The emulsifying and gelling properties of pectin-based emulsion gels are intimately linked to the molecular architecture of pectin. Complex structural parameters—including molecular weight (Mw), protein content, degree of acetylation, degree of methoxylation (DM), ferulic acid content, galacturonic acid (GalA) proportion, methyl-ester distribution, degree of branching, and amidation—collectively determine interfacial adsorption, network formation, and overall colloidal stability (Jia et al., 2024; Ngouémazong et al., 2015).

With respect to emulsification, low Mw produces thinner adsorbed layers and weaker steric stabilisation, whereas higher Mw supports stronger intermolecular interactions and improved long-term stability (W. X. Jiang et al., 2021). Proteinaceous moieties enable effective adsorption at the oil–water interface, lowering interfacial tension and inhibiting droplet aggregation, flocculation, and phase separation (Akhtar et al., 2002; Funami et al., 2007). The influence of acetyl content is complex and source-dependent. Deacetylation reduces the emulsifying activity of citrus pectin (Dea & Madden, 1986), whereas chemical acetylation improves it (Leroux et al., 2003). In contrast, beet pectin is far less sensitive to deacetylation, indicating that the contribution of acetyl groups varies with pectin origin. Ferulic acid content may further influence emulsification, as it was observed that the adsorbed pectin fraction at the interface contains a higher ferulic acid content than the non-adsorbed fraction(Siew & Williams, 2008); nevertheless, a direct causal relationship has yet to be conclusively established. DM also modulates interfacial and emulsifying properties, although its exact effects remain a subject of ongoing debate (Jia et al., 2024; C. Wang, Qiu, et al., 2021). DM modulates interfacial behaviour through two opposing contributions: high DM favors hydrophobic anchoring but reduces charge density, whereas low DM strengthens electrostatic stabilisation (Alba & Kontogiorgos, 2017; Ngouémazong et al., 2015). Which contribution dominates is determined by the instantaneous electrostatic environment (pH, ionic strength and the presence of protein) and by the availability of alternative stabilising motifs (RG-I side chains, residual proteinaceous moieties and acetyl groups). Consequently, high-DM pectins often outperform low-DM pectins in protein-poor, low-salt systems, while low-DM pectins excel in protein-containing or moderately acidic systems (Alba & Kontogiorgos, 2017). Environment-dependent effects of DM on emulsifying performance are summarised in Table 3. Future comparative studies must report the full set of co-variables—pH, ionic strength, protein content, Mw and side-chain profile—rather than DM in isolation; only then can a quantitative structure–function map be constructed.

**Table 3:** Environment-dependent effects of degree of methoxylation (DM) on pectin emulsification.

**Regulating variable**	**Conditions favouring high DM**	**Conditions favouring low DM**	**Mechanistic interpretation**
pH	Near-neutral to mildly acidic (pH ≈ 5–7), protein-poor systems	Strongly acidic (pH < 3.5) or near the protein isoelectric point	At higher pH, carboxyl groups are dissociated; high charge density of low-DM pectin can hinder close packing, whereas hydrophobic methyl esters of high-DM pectin support anchoring. At low pH, protonation reduces electrostatic repulsion and hydrophobic contacts become more decisive
Presence of protein	No protein or very low residual protein	Residual or added protein	Protein adsorbs first; low-DM pectin can form a stronger secondary layer by electrostatic complexation near the protein pI. Without protein, hydrophobic anchoring by high-DM pectin is often rate-limiting
Ionic strength	Low salt	Moderate to high salt	Salt screens charge and diminishes the electrostatic advantage of low-DM pectin, so the hydrophobic contribution of high DM becomes relatively more important
Homogenization energy and molecular weight	Mild homogenization that preserves higher Mw	Intense homogenization that partially depolymerises chains	High-energy processing alters chain length and side-chain bulk; the net benefit of high versus low DM then depends on how much steric corona and charge density remain after degradation
RG-I side-chain content	Low RG-I branching	Higher RG-I branching	Branched neutral-sugar side chains add steric stabilisation and can partly offset weak hydrophobic anchoring of low-DM pectin; when branching is low, the hydrophobic contribution of DM is more critical
Oil polarity / oil-volume fraction	Formulation-dependent; often discussed for more polar oils or demanding high-oil systems	Formulation-dependent; lower-polarity oils or moderate oil fractions	Oil polarity and volume fraction change the efficiency of hydrophobic group orientation and the relative need for steric versus electrostatic stabilisation; reported trends remain system-specific

*Note.* Compiled from Akhtar et al. (2002); Alba and Kontogiorgos (2017); Funami et al. (2007); Huang et al. (2025); Ngouémazong et al. (2015); Qi et al. (2024); Wang, Qiu, et al. (2021); Yan et al. (2021). Outcomes are formulation-dependent.

The gelation properties of pectin are equally governed by its diverse structural features. For high-methoxy pectin (HMP), a lower GalA content reduces the number of carboxyl groups, thereby decreasing electrostatic repulsion between chains and promoting additional hydrogen bonding that contributes to network formation. In LMP, higher GalA content combined with greater Mw provides longer chains and more metal-ion binding sites, which favor the development of robust gel networks (Cao et al., 2020). It has been established that pectin with higher Mw exhibits faster gelation kinetics and higher elastic modulus, whereas very low Mw (≤ 10 kDa) can result in a complete loss of gel-forming ability. For HMP, lower DM reduces the number of methoxyl groups and consequently weakens hydrophobic interactions, which are unfavorable for gel formation. Conversely, for LMP, lower DM increases the number of free carboxyl groups available for ionic bridging, accelerating gelation. Thus, the gelation rate of HMP increases with rising DM, while that of LMP decreases with rising DM. The distribution pattern of methyl esters also plays a decisive role: the homogalacturonan (HG) region can be divided into methylated and non-methylated domains. Methylated domains participate in hydrophobic interactions and hydrogen bonding but cannot bind Ca^2+^ or be cleaved by polygalacturonase, whereas non-methylated domains readily form Ca^2+^ cross-links and are susceptible to enzymatic cleavage. Consequently, a high proportion of methylated domains favors gel formation in HMP, while a high proportion of non-methylated domains favors gel formation in LMP (Cameron et al., 2015). The degree of branching exerts conditional effects. In HMP, neutral sugar side chains on the branches can either hinder or enhance gel cohesion through hydrophobic interactions, depending on the specific molecular geometry and interaction with surrounding water molecules (Lara-Espinoza et al., 2018; Oakenfull & Scott, 1984). In general, higher branching introduces greater steric hindrance to Ca^2+^ binding, which is unfavorable for LMP gelation; however, in certain LMP structures where hydrogen bonding is the dominant force, high branching can promote inter-chain hydrogen-bond formation and thereby facilitate gelation (S. G. Chen et al., 2021). Finally, amidation increases the potential for hydrogen bonding between pectin chains and therefore enhances gel-forming ability (Lootens et al., 2003).

In practice, absolute values of these intrinsic parameters are shaped by botanical origin, extraction conditions and subsequent modification, which therefore constitute important levers for tuning the interfacial and gelling behaviour of pectin. Pectin obtained from different plant sources displays distinct structural profiles. Based on a comparison of pectin from tomato, carrot, onion, and apple, tomato pectin was found to possess the highest molar mass, carrot pectin to exhibit higher branching but lower molar mass, and onion pectin to be characterized by elevated glucose and galactose contents together with the lowest molar mass (Neckebroeck et al., 2020). Apple pectin was noted to generally yield gels with superior elasticity and viscosity, citrus pectin to produce more elastic yet brittle gels, and sugar-beet pectin to show relatively poor gelling performance (Chan et al., 2017). Extraction methods exert a similarly pronounced influence. The three primary categories—chemical (acid or alkali), physical (microwave, ultrasound, steam explosion, ion-exchange), and biological (enzymatic or microbial)—differentially affect the final structure. Acid extraction is typically employed to obtain high-viscosity HMP, while alkali extraction is preferred for LMP or amidated pectin because it better preserves neutral sugar side chains (Wandee et al., 2019; W. J. Wang et al., 2016). Alkali combined with cellulase extraction of citrus pectin was demonstrated to yield the highest RG-I content (61.80%), the smallest particle size, and the most compact network structure (Cui et al., 2020). Pectin is also frequently modified—through physical (ultrasound, high pressure, shear, plasma, microwave), chemical (esterification, etherification, amidation, acetalization, depolymerization, cross-linking), or enzymatic methods (HG- or RG-I-specific enzymes such as pectin methylesterase, lyase, polygalacturonase, and acetyl esterase)—to tailor its structure for specific applications (D. Q. Li et al., 2021; Yue et al., 2023). Through multifactorial analysis of okra pectin treated with combinations of glycoside hydrolases, it was concluded that while Mw and viscosity influence emulsification, neutral sugar side chains (arabinogalactan, galactan, arabinoglycan) are the primary determinants of long-term emulsion stability (Olawuyi et al., 2022). Phenylalanine-amidated pectin prepared via low-temperature enzymatic modification was shown to exhibit superior cream index and emulsion stability compared with native pectin and low-amidated controls, effectively preventing premature oil–water separation and enhancing curcumin bioaccessibility (C. M. Zheng et al., 2023).

These individual structural features do not operate in isolation. Their relative contributions are continuously re-weighted by environmental conditions, most notably pH. High-Mw chains adsorb more slowly; when pH reduces net charge or promotes self-association, the kinetic penalty becomes severe (Ngouémazong et al., 2015). At neutral pH the hydrophobic contribution of DM and acetyl groups is often secondary to electrostatic effects, whereas under acidic conditions protonation of carboxyl groups allows hydrophobic interactions to dominate (Wang, Qiu, et al., 2021). Protein content illustrates the most pronounced pH-dependent interaction: above the protein isoelectric point both components are anionic and complexation is limited; near or below the pI the protein becomes cationic, triggering rapid electrostatic complexation that accelerates adsorption and strengthens the interfacial film (Akhtar et al., 2002; Funami et al., 2007).

Side-chain architecture interacts with all of the above. Beyond the mere presence of arabinose and galactose residues, the length, degree of branching and specific configuration of RG-I side chains critically determine interfacial performance. Linear or sparsely branched arabinan and galactan chains of moderate length increase the hydrodynamic thickness of the adsorbed layer and enhance steric repulsion. Highly branched Type-II arabinogalactans create a more voluminous, hydrated corona that elevates dilatational elasticity and viscosity, retarding coalescence (Bindereif et al., 2021; Niu, Wang, et al., 2024). The relationship is non-monotonic: excessively long or dense side chains can slow adsorption kinetics, whereas controlled enzymatic trimming to an intermediate length optimises both rapid anchoring and robust steric stabilisation (El Omari et al., 2022). RG-I-rich fractions therefore form more elastic interfacial films than HG-dominant counterparts and can stabilise emulsion gels even at reduced calcium levels (Qi et al., 2024).

Collectively, the emulsifying performance and gel-network-forming ability of pectin are linked through a continuous multiscale sequence: molecular architecture → interfacial adsorption kinetics and film structure → continuous-phase network topology → macroscopic rheology and stability. Interfacial dilatational and shear rheology provide the decisive experimental window onto this sequence. Molecular weight and chain linearity control the ability of pectin to reach the interface; proteinaceous and feruloyl moieties supply the primary hydrophobic anchors, yet their accessibility is modulated by the surrounding carbohydrate matrix; RG-I side-chain architecture simultaneously governs hydrated-layer thickness, dilatational elasticity and continuous-phase entanglement; and DM and its distribution create trade-offs between hydrophobic anchoring and electrostatic stabilisation. Any attempt to engineer pectin emulsion gels solely by adjusting DM or Ca^2+^ concentration is therefore incomplete. The oil–water interface and the continuous-phase network must be treated as a single, structure-coupled continuum. Only quantitative mapping of how each molecular feature simultaneously affects interfacial film viscoelasticity and bulk network topology will yield truly predictive structure–interface–function relationships.

### 4.2 External environmental factors

In addition to the intrinsic structural characteristics of pectin, external environmental factors exert a substantial influence on the textural, structural, and functional properties of pectin-based emulsion gels. The principal external factors include the homogenization method, the type and concentration of the oil phase, the type of emulsifier, the type and ratio of the gel matrix, and the type and concentration of the gel inducer. These external conditions constantly interact with the aforementioned pectin structure.

The homogenization emulsification method significantly affects the network structure and functional properties of the resulting gels. In most studies, preparation begins with the formation of a homogeneous and stable emulsion by combining the oil and aqueous phases using high-shear, high-pressure, or ultrasonic methods. Different homogenization techniques produce distinct gel microstructures and inter-droplet interactions, which in turn modulate mechanical strength and release behaviour (Geng et al., 2022; Hou et al., 2016). For example, ultrasound-treated emulsion gels exhibit markedly smaller droplet sizes, higher water-holding capacity, a more homogeneous and compact network, and improved chemical stability and bioaccessibility of encapsulated compounds such as β-carotene compared with untreated controls (Zhang et al., 2021).

The type of oil phase and its addition level also play a decisive role in the physicochemical properties of pectin-based emulsion gels. The influence of different oils (olive oil, peanut oil, and 24° palm oil) and oil-phase fractions (40–80% *v*/v) on the apparent properties, rheology, texture, and microstructure of transglutaminase-induced peanut protein–pectin composite emulsion gels was systematically examined, identifying 60% 24° palm oil as the optimal condition for superior gel performance (Ding et al., 2022). RG-I pectin emulsion gels prepared with five oil types (soybean oil, corn oil, sunflower oil, peanut oil, and medium-chain triglycerides) were found to exhibit that soybean oil yielded the highest gel strength and the most effective colon-targeted delivery of nobiletin (Qi et al., 2024). Increasing emulsion concentration (5–30 wt%) was further demonstrated to enhance swelling capacity; at 10 wt%, the gels displayed interconnected pores and optimal hardness, adhesiveness, and chewiness (L. Feng et al., 2019). Collectively, these findings indicate that an appropriate oil-phase type and concentration are critical for optimizing texture and microstructure.

The type of emulsifier strongly influences the rheological properties, texture, and water-holding capacity of the gels. Four emulsifiers—whey protein hydrolysate (WPH) and its succinylated (W—S), glycated (W-L), and dual-modified (W-S-L, W-L—S) derivatives—were compared, with the modified variants shown to provide stronger electrostatic repulsion and steric stabilisation. Consequently, the resulting emulsion gels exhibited higher viscosity, denser network structures, restricted oil-droplet mobility, and markedly improved gelation properties. In particular, the W-S-L stabilised gel achieved the highest curcumin bioaccessibility (65.57%) (Pan et al., 2021).

The type and ratio of the gel matrix also exert a pronounced effect. In general, composite matrices formed by combining polysaccharides and proteins yield denser networks, stronger interfacial activity, and superior encapsulation efficiency compared with single-polysaccharide or single-protein systems. Enzymatic gels prepared using sugar-beet pectin (SBP), soybean protein isolate (SPI), or SBP/SPI complexes as matrices demonstrated that the composite matrix exhibited higher interfacial uptake, denser networks, better encapsulation, and slower aroma release than the individual components (Hou et al., 2016). Adjusting the matrix ratio offers a practical means of tuning gelation behaviour. Varying SBP/SPI ratios under laccase- and heat-induced gelation was evaluated, and it was found that mechanical and thermal stability increased with higher SBP content and decreased with higher SPI content (L. Feng et al., 2021). The impact of pectin addition on pectin–soybean protein isolate gels was investigated, and it was observed that pectin concentration had little effect on hardness but caused elasticity to first increase and then decrease, while the storage stability of embedded vitamins (V_E_ and D‑sodium isoascorbate) progressively declined (Mu et al., 2018). These contrasting results likely stem from differences in pectin source and structural type.

Finally, the type and concentration of gel inducers are key determinants of network structure. Previous studies have shown that SPI-SBP emulsion gels prepared using dual induction (laccase + transglutaminase and laccase + GDL) exhibit a denser structure with higher cross-linking density and fewer pores due to covalent bonding within and between protein and pectin molecules. GDL is a widely used acidulant that gradually lowers pH to induce HMP gelation or controls Ca^2+^ release in LMP systems (Zhang, Zheng, et al., 2022). Examination of GDL concentrations (0–2.0% *w*/w) in pectin-based emulsion gels identified 1.6% as optimal for the highest storage modulus and excellent 3D-printing performance (smooth surface, structural integrity, and high resolution) (J. Li et al., 2023). Incorporation of Ca-EDTA (0.4 mol/L) and GDL (0.6 mol/L) into LMP emulsions revealed that increasing Ca^2+^ content (40–200 mg/g) continuously enhanced gel strength and elasticity by increasing ion-binding regions and cross-linking density in the aqueous phase (Yang et al., 2022).

Importantly, these external variables should not be varied in isolation from pectin structure. The same homogenization energy, oil fraction or inducer dose can produce different outcomes depending on DM, RG-I branching and protein content. The dispersed oil phase also participates in network assembly, so hydrogel-derived structure–property rules are incomplete for emulsion gels. Future work should therefore map external conditions together with pectin fine structure rather than as independent factors.

## 5 Applications

The principal application areas are illustrated in Fig. 4. Pectin-based emulsion gels have found uses in food structuring, bioactive delivery, edible coatings and packaging, and 3D food printing, with further interest in nutraceutical and pharmaceutical systems. The combination of a dispersed oil phase and a gelled continuous phase supports texture design, protects lipophilic compounds such as curcumin, nobiletin, β-carotene and flavour esters from oxidation, heat and light, and can enable controlled release. Printable inks and, still at an early stage, specialised cleaning formulations have also been proposed.

**Fig. 4:**
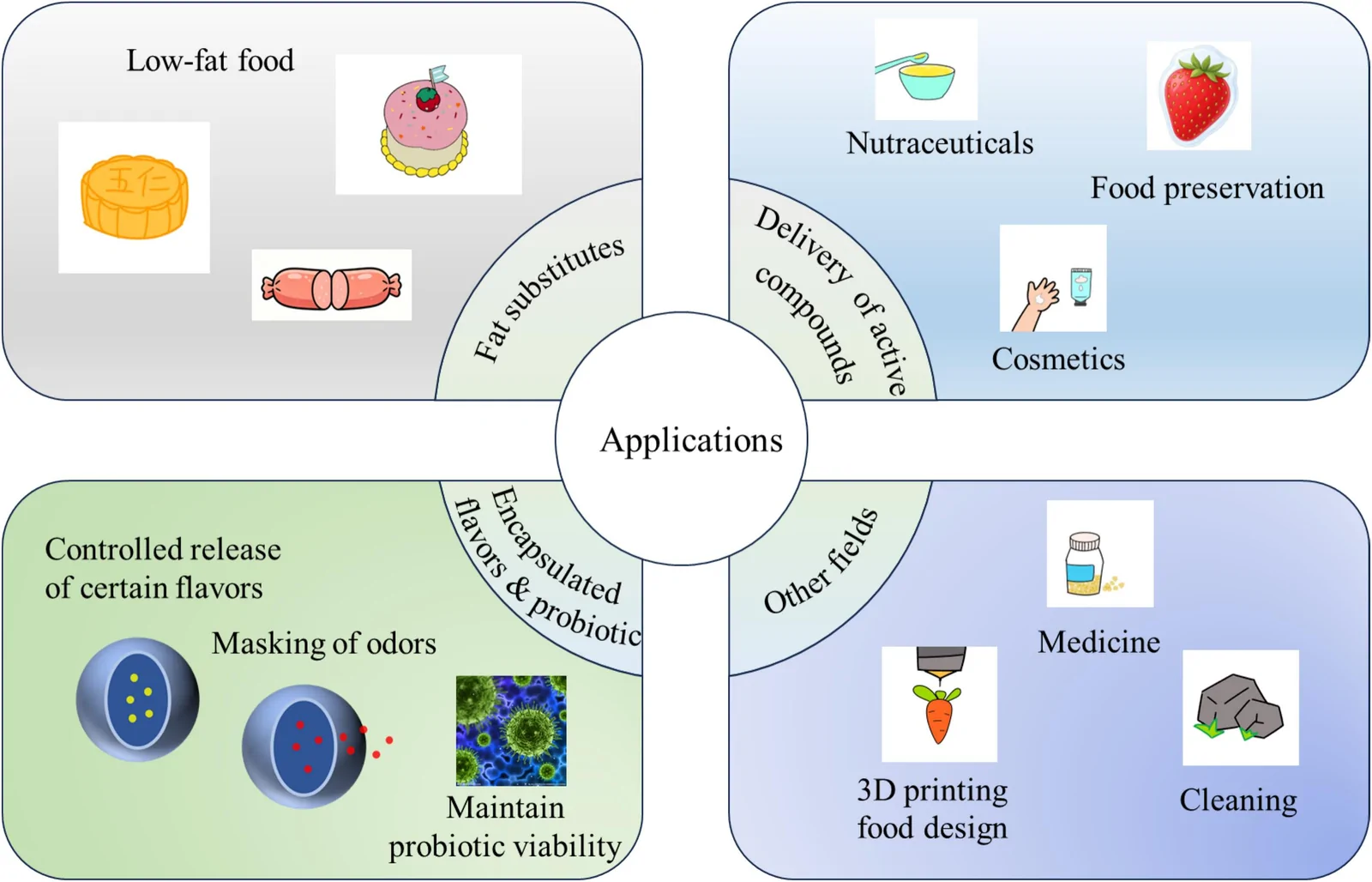
Applications of pectin-based emulsion gels.

Performance in these settings depends not only on process conditions and pectin structure, but also on whether the system is built from pectin alone or from a pectin–protein composite. Pure pectin emulsion gels suit clean-label, low-protein or allergen-conscious products where formulation simplicity matters. Composite systems are usually preferred when high oil levels, heat or freeze–thaw stress, or simultaneous structuring and delivery are required—as in fat replacement in meats and bakery goods, co-encapsulation of hydrophilic and lipophilic actives, and matrices that must survive processing or gastrointestinal transit. The following subsections follow this distinction where it is relevant to the application. For reference, Table 4 summarises representative pectin-based emulsion gels reported in the literature, listing their substrate composition and structural features, emulsification and gelation routes, dominant intermolecular forces, principal characterization methods and intended applications.

**Table 4:** Preparation, formation mechanism, characterization and application of pectin-based emulsion gels.

Substrate type	Structural features of substrate	Emulsifier/Oil phase type and content	Emulsification processing conditions	Gel induction methods	Main force	Characterization	Applications	References
Pectin	Pectin from the seeds of *Nicandra physalodes* (Linn.) Gaertn. (NPGS)	-/Walnut oil	High–speed shear homogenization (HSSH) under 12,000 rpm for 2 min	0%, 0.4%, 0.8%, 1.2%, 1.6% and 2.0% of Glucono–delta–lactone (GDL) (*w*/w)	Hydrogen bonding, Van der Waals forces	Rheological properties, Confocal laser scanning microscopy (CLSM), gel strength	Encapsulation of anthocyanin and three–dimensional printing (3D printing)	(Li et al., 2023)
High-methoxy pectin (HMP)	HMP (no details)	-/Corn oil	High-pressure homogenization (HPH) three times at 100 MPa and HSSH 8000 rpm for 2 min	Ethanol-induced pectin gelling	Hydrogen bonding, Van der Waals forces and hydrophobic interactions	Rheological properties, particle size, CLSM	Improvement of the bioaccessibility and reduction of irritancy of capsaicin	(He et al., 2023)
	HMP (derived from citrus, DM > 50%): Soybean protein isolate (SPI) (3:7)	−/6% (*v*/v) soybean oil	HSSH at 20,000 rpm for 5 min，then ultrasound homogenization (UH) at different output powers (0, 150, 300, 450 and 600 W) at 20 kHz for 15 min (pulse duration of 4 s on and 2 s off)	Thermal induction: the emulsion is heated at 80 °C for 30 min, then cooled in an ice bath	Hydrogen bonding, Van der Waals forces and hydrophobic interactions	CLSM, scanning electron microscopy (SEM), water holding capacity, low field nuclear magnetic resonance (LF-NMR), differential scanning calorimetry (DSC), gel strength	Encapsulation of β-carotene for improved stability and bioaccessibility of β-carotene	(Zhang et al., 2021)
Low-methoxy pectin (LMP)	Four LMP (DM = 33.3%, 27.2%, 37.1%, 41%)	Tween 60/Virgin olive oil	HSSH 2000 rpm for 2 min	18% (*w*/w) calcium to pectin	Hydrogen bonding, hydrophobic interactions, ionic bond	Rheological properties, optical microscopy	Potential cosmetic applications	(Lupi et al., 2015)
	LMP (DM = 45.40%, 15.06%)	Tween 20/Soybean oil	HSSH 13,000 rpm for 4 min, HPH at 50 MPa thrice for 3 min	CaCO_3_ (0.5 wt%), GDL (0.8 wt%)	Ionic bond	Swelling ratio, SEM, gel strength, rheological properties	Encapsulation and sustained release of curcumin	(Zhang, Zheng, et al., 2022)
	LMP (DM = 25.84%)	−/10% soybean oil	15% power for 3 min (open 10 s and stop 5 s) and then UH at 40% power for 10 min (over 2 s and stop 2 s)	Ca-EDTA (0.4 mol/L, 120 mg/g Ca^2+^), GDL (0.6 mol/L)	Ionic bond	Texture profile analysis (TPA, gel strength and gel elasticity), LF-NMR, CLSM	Encapsulation and sustained release of curcumin, low-calorie model	(Yang et al., 2022)
	LMP (DM: 15.27%, β: 0.02, Mw: 2.006 × 105 g/mol) derived from citrus physiological premature fruit drop	−/10% soybean oil, peanut oil, corn oil, sunflower oil and medium-chain triglyceride	HSSH 10,000 rpm for 3 min, and then UH for 5 min (2 s sonication, 2 s stop) at 30% energy	30 mg/g Ca^2+^	Ionic bonds through calcium bridges between two carboxyl groups of adjacent pectin chains	LF-NMR, CLSM, gel strength, rheological properties	Encapsulation and colon targeted delivery of nobiletin	(Qi et al., 2024)
Pectin-protein	Sugar beet pectin (SBP) + SPI	−/20% (w/w) medium-chain triglyceride	HSSH 10000 rpm for 3 min，then HPH five times at 30 MPa	Laccase and glucono-δ-lactone or laccase and transglutaminase, heating the emulsion at 40 °C for 4 h	Covalent bonding between SBP intra- and inter-molecularly	TPA, SEM, CLSM, Swelling ratio	Encapsulation of β-carotene and riboflavin	(Zhang, Yin, et al., 2022)
	SBP + SPI	2 wt% Tween 20/20 wt% Medium-chain triglyceride oil	HSSH at 11,000 rpm for 3 min，then HPH at 600 bar	Laccase-induced cross-linking reaction (at 40 °C for 2 h), followed by heat treatment (heating at 90 °C for 30 min and then cooling to room temperature)	Covalent cross-linking among phenolic groups and amino acids	TPA, water holding capacity, SEM thermo-gravimetric analysis, Rheological properties,	Potential design and application in the food industry	(L. Feng et al., 2021)
	HMP + SPI	−/ 50 wt% soybean oil	HSSH at 13,000 rpm for 2 min	Final concentration of Ca^2+^ is 0.04 g/mL, mixture was heated to 85 °C for 25 min	Strong protein–pectin interfacial complexation	Rheology, TPA, microstructure, sensory evaluation	Fat replacer in pork sausage (improved texture & quality)	(A. Zhang et al., 2025)
	Pea protein isolate +HMP	−/20–60 wt% Soybean oil, peanut oil, and olive oil	HSSH 13,000 rpm for 2 min	Transglutaminase treatment at 40 °C for 2 h, followed by heating at 85 °C for 30 min.	Electrostatic & hydrophobic interactions	Rheology, TPA (hardness, adhesiveness), microstructure	Plant-based whipped cream / fat substitute	(Chen et al., 2025)
	SBP (DM = 56.5%, protein and ferulic acid contents were 56 and 9 g kg^−1^) + SPI	-/Corn oil	HSSH 10,000 rpm for 2 min, then HPH twice at 300 bar	Microbial transglutaminase	Covalent cross-linking between proteins adsorbed on different droplets	Size, CLSM	Controlled slow release of aroma (ethyl butyrate, ethyl hexanoate and ethyloctanoate)	(Hou et al., 2016)

### 5.1 Fat substitutes

Emulsion gels serve as intermediate materials between liquids and solids, effectively mimicking the sensory and physical attributes of saturated fats while offering a healthier alternative. They are therefore increasingly explored as fat replacers in dairy, meat, and bakery products to reduce risks associated with obesity, type 2 diabetes, and cardiovascular disease (Liu et al., 2023).

Early applications already demonstrated the potential of pectin: banana pectin successfully replaced 30% of the fat in salad cream (Maneerat et al., 2017), and citrus high-methoxy pectin (HMP) was used to formulate low-fat mooncake fillings (Xu et al., 2022). More recent work has shifted toward structured pectin-based emulsion gels. Pectin coatings on whey-protein-stabilised nanoemulsions progressively lowered lipid digestibility as the degree of methoxylation (and thus charge density) decreased (Kadiya & Ghosh, 2022). Passion-fruit-pectin emulsion gels have been characterized rheologically and structurally as a foundation for sustainable fat-replacement strategies (Kavya et al., 2023). Hybrid Pickering systems that combine pectin with zein nanoparticles and hydroxypropyl guar gum further yield thermally stable gels suitable as functional fat substitutes (Sun et al., 2026).

In real meat products, soy-protein–HMP composite emulsion gels have replaced 25–100% of pork back fat in sausages. At 25% replacement, hardness, chewiness and overall sensory acceptability remained comparable to the full-fat control, while in-vitro protein digestibility improved (Zhang, Yang, et al., 2025). In bakery systems, high-methoxy citrus pectin gels incorporated into moon-cake skins reduced fat content from ∼22% to ∼14% while preserving water-holding capacity and sensory quality (Xu et al., 2022).

These product-level trials confirm that pectin emulsion gels can function as practical fat replacers. Nevertheless, matching the melting profile, flavour release and long-term oxidative stability of conventional animal fats and shortenings remains a challenge, and most studies are still limited to laboratory- or pilot-scale sensory and rheological evaluation rather than large-scale nutritional validation.

### 5.2 Encapsulation and delivery of bioactive compounds

Beyond their role as food ingredients, pectin-based emulsion gels are widely investigated for the encapsulation and delivery of bioactive substances—including probiotics, flavors, vitamins, and pharmaceuticals—across food, packaging, nutraceutical, and medical fields. Unlike simple hydrogels, the presence of an oil phase makes emulsion gels particularly effective for protecting and delivering lipophilic compounds (Mao et al., 2020).

#### 5.2.1 Protection and sustained release of probiotics

For probiotics, pectin acts as both a structural matrix and a prebiotic, enhancing survival through the upper gastrointestinal tract. Synbiotic systems combining probiotics with pectin have shown high encapsulation efficiency and improved storage stability (Gao et al., 2022; Raddatz et al., 2020). Recent studies confirm that double-network pectin emulsion gels (formed with GDL and calcium) provide strong thermal protection and inhibit contact with gastric acid and bile salts, thereby enhancing gastrointestinal viability (S. X. Huang et al., 2025). It was reported that pickering emulsion gels stabilised by milk fat globule membrane/pectin significantly enhanced probiotic viability under thermal and gastrointestinal stress(Ji et al., 2025).

#### 5.2.2 Controlled release and retention of flavour compounds

Flavour encapsulation benefits from the dense network, which enables slow or controlled release and masking of undesirable odors. Substantially higher retention of ethyl acetate and linalool was reported in emulsion gels (60–100%) compared with non-gelled emulsions (5–25%) (Lee et al., 2006). Compact interfacial networks in SBP/SPI gels were further demonstrated to significantly reduce ethyl butyrate release (Hou et al., 2016). Protein–pectin delivery carriers have been comprehensively reviewed, highlighting that the strong interfacial interactions and gel network formation enable effective encapsulation and controlled release of volatile bioactive compounds (Wen et al., 2025).

#### 5.2.3 Encapsulation of lipophilic bioactive compounds

Loading lipophilic bioactive compounds such as curcumin, nobiletin and carotenoids—known for their anticancer, anti-inflammatory, antioxidant and antidiabetic properties—into pectin-based emulsion gels markedly improves their stability, digestive protection and controlled release (Y. Y. Chen et al., 2023; Stohs et al., 2020). These compounds are typically poorly water-soluble, sensitive to light, heat and ions, and exhibit low oral bioaccessibility when administered in free form (Epstein et al., 2010; Tian et al., 2009). Zhang, Zheng, et al. (2022) successfully encapsulated curcumin within the oil phase of an RG-I-enriched pectin emulsion gel, achieving effective protection and sustained release. The same group also developed a novel SPI–SBP emulsion gel for the co-encapsulation of hydrophilic (riboflavin) and lipophilic (β-carotene) nutrients (Zhang, Yin, et al., 2022). Another study further demonstrated that different LMP significantly affect curcumin loading and colon-targeted release in emulsion gels. Using high hydrostatic pressure (HHP)-treated LMP compared with alkaline de-esterified LMP (DM 5–10%), they achieved an encapsulation efficiency of 92.55 ± 2.19%. The HHP-pectin gels exhibited a denser, more uniform network structure, markedly enhancing light and thermal stability of curcumin (retention rates of approximately 70.91% after UV exposure and 79.91% after thermal treatment). These gels effectively inhibited curcumin release in the oral, gastric, and small-intestinal phases while providing superior sustained release in the colon (89.03 ± 0.76% at the same DM range), attributed to higher molecular weight and viscosity that strengthened the Ca^2+^-bridged network (Arain et al., 2025). Ethanol-induced pectin gelation was employed to encapsulate capsaicin, which significantly reduced gastrointestinal irritation while enhancing its bioaccessibility and therapeutic effects (anticancer, antioxidant, anti-inflammatory, and metabolic regulation) (J. X. He et al., 2023).

#### 5.2.4 Nutraceutical oral delivery

For oral nutraceutical delivery, pectin-based emulsion gels provide a convenient, plant-derived alternative to traditional liquid emulsions, bulk oils, or capsules, addressing the needs of children, the elderly, and patients with swallowing difficulties. Gelatin-based emulsion gels have been widely studied for their excellent emulsifying capacity and texture; however, concerns regarding their animal origin (ecological, ethical, and health issues) and low melting temperature (leading to thermal instability) have driven the development of plant-based systems (Baydin et al., 2022; Dille et al., 2018; Hattrem et al., 2015). Polysaccharide emulsion gels containing pectin and agar were prepared and compared directly with gelatin-based counterparts. Rheological analysis revealed similar gelling and melting temperatures (38 °C and 54 °C), while texture analysis showed that the pectin-based gels were softer, allowing formulation flexibility according to market requirements. In vitro lipolysis experiments indicated that pectin-based gels exhibited a slower initial lipolysis rate (4.3 μmol FFA/s) and lower overall degree of lipolysis compared with gelatin gels (24.9 μmol FFA/s), suggesting potential for controlled release, although the reduced lipolysis may also lower absorption efficiency in some cases (Baydin et al., 2022).

#### 5.2.5 Edible coatings and active food packaging

Pectin-based emulsion gels are well suited for edible coatings and active packaging aimed at food preservation. They combine a continuous hydrophilic network with a dispersed oil phase that can carry lipophilic antimicrobials and antioxidants. The resulting films or coatings regulate moisture and gas exchange, delay ripening and extend the shelf life of perishable fruits, vegetables and minimally processed foods, while reducing reliance on disposable plastic packaging (Muñoz-Labrador et al., 2018; Pholsin et al., 2024; Ye et al., 2019). Although single pectin coatings are often monofunctional, incorporation of bioactive compounds markedly enhances preservation performance. Two natural antimicrobials—*Biosecur F440D* (a citrus extract) and essential oils—were encapsulated in pectin-based emulsion gels; the systems slowed antimicrobial release, improved storage stability and effectively inhibited *Listeria monocytogenes* and *Penicillium chrysogenum* on pre-cut carrots, thereby prolonging shelf life (Ben-Fadhel et al., 2020). Collectively, pectin emulsion gels coatings function simultaneously as packaging materials and preservation agents. Remaining challenges include matching the mechanical strength and moisture resistance of synthetic films under high-humidity storage, ensuring consistent active-compound release over the intended shelf life, and scaling coating processes for industrial produce lines. Addressing these issues will be essential for translating laboratory coatings into commercially viable, clean-label food-preservation solutions.

#### 5.2.6 Key limitations and future perspectives

It must be emphasised, however, that the majority of current evidence is derived from in-vitro simulated digestion experiments. In these models, pectin-based emulsion gels frequently demonstrate high encapsulation efficiency, protection against gastric degradation and improved micellarisation or intestinal release—collectively reported as enhanced bioaccessibility. Bioaccessibility measured in vitro is not synonymous with bioavailability in vivo. True oral bioavailability encompasses intestinal absorption, first-pass metabolism, systemic distribution and elimination—processes that static or semi-dynamic digestion models cannot fully capture. At present, animal or human pharmacokinetic data specifically for pectin-based emulsion gels remain extremely scarce. The few available in-vivo studies that incorporate pectin—typically as protein–pectin nanoparticles or related nano-carriers rather than true emulsion gels—suggest possible improvements in systemic exposure. For example, pectin–sodium caseinate nanoparticles co-encapsulating resveratrol and curcumin increased plasma resveratrol levels and raised maximal tissue concentrations by 0.83–1.27-fold in mice relative to resveratrol-only nanoparticles (Zhou et al., 2026). Curcumin nanosuspension encapsulated in chitosan–pectin microbeads likewise produced a 2.5-fold increase in C_max_ and a 4.4-fold increase in AUC_0–48h_ compared with free curcumin in animal models (Sampathi et al., 2023). These systems differ in structure and composition from the emulsion gels discussed in this review, and their number remains too limited to support general claims for pectin-based emulsion gels. Consequently, statements that pectin emulsion gels “improve bioavailability” should be restricted to cases where actual in-vivo pharmacokinetic evidence exists. For the bulk of the literature, the more accurate description is that these systems improve in-vitro digestive stability and bioaccessibility. Bridging the gap between promising in-vitro release profiles and demonstrated in-vivo bioavailability—through well-designed animal pharmacokinetic studies and, ultimately, human trials—constitutes one of the most urgent tasks for the field.

These studies have substantially advanced our understanding of the delivery behaviour of pectin-based emulsion gels and have provided valuable guidance for the development of functional food, nutraceutical, pharmaceutical, and packaging materials with precisely controlled release profiles. However, the full industrial and clinical potential of these systems has yet to be realized and warrants continued exploration.

### 5.3 3D printing

3D printing technology is attracting growing interest in the food industry for its ability to create customized shapes and textures (T. Feng et al., 2022). Emulsion gels serve as excellent printable inks because they combine emulsion stability during storage with protection of encapsulated actives against temperature and pH fluctuations (Farjami & Madadlou, 2019; Komaiko & McClements, 2015). The structural integrity and tunable rheology of pectin-based emulsion gels make them particularly suitable for precise food design. Anthocyanin-loaded pectin–GDL emulsion gels with favourable rheology, storage stability and printability have been used as color inks for cookies, rice cakes and related products (J. Li et al., 2023). Fucoxanthin-loaded Pickering emulsion gels stabilised by salmon-protein/pectin complexes achieved high print fidelity together with a 1.37–1.92-fold improvement in bioaccessibility (Zhong et al., 2024). Pectin–medium-chain-triglyceride oleogel pastilles loaded with curcumin have likewise been 3D-printed into ready-to-eat formats with acceptable texture and elevated intestinal bioaccessibility (Negi & Moses, 2025)**.** Plant-based emulsion gels for 3D-printed foods have been reviewed, highlighting pectin's role in tailoring printability and texture (Liu, Cheng, et al., 2025). Nevertheless, 3D food printing technology is not yet mature for large-scale production. Challenges remain in matching the melting profile and flavour release of conventional products, ensuring long-term post-print stability and oxidative resistance, and scaling extrusion processes while retaining shape fidelity. Systematic evaluation of sensory acceptance, storage behaviour and process economics under realistic production conditions will be essential for industrial translation.

### 5.4 Cosmetic applications

Emulsion gels (primarily oil-in-water systems) also offer considerable potential in cosmetics owing to the complementary advantages of emulsions (controllable rheological properties, reduced greasiness, and good skin penetration) and hydrogels (ease of application and removal, compatibility with a wide range of excipients, and a three-dimensional spatial structure). Low-methoxy pectin (LMP) can form gels at room temperature, making it especially suitable for encapsulating thermally unstable ingredients without causing degradation (Awasthi, 2011; Munarin et al., 2012). It has been reported that emulsion gels can be prepared using four different LMPs as the matrix with calcium chloride addition, and that the formulation can be optimized by systematically varying the pectin mass fraction and oil content to achieve rheological properties suitable for cosmetic applications (Lupi et al., 2015).

### 5.5 Cleaning and other emerging applications

Pectin-based emulsion gels also show promise in cleaning applications, particularly for cultural-heritage preservation. Cavallaro et al. (2019) demonstrated that Pickering emulsions within a pectin-gel matrix effectively remove wax layers from marble surfaces, with cleaning efficacy controlled by contact time. This green approach offers a sustainable alternative to traditional solvents. To date, however, literature on cleaning applications remains scarce, with most research focused on food and delivery uses. Further exploration is needed to broaden pectin-based emulsion gels into non-food fields.

Despite promising laboratory performance, the translation of pectin-based emulsion gels into commercial products or clinical applications remains limited. Most studies are still at the proof-of-concept or in vitro stage, with insufficient validation of long-term stability, scalability, and in vivo efficacy under realistic food matrices or delivery conditions. Fat substitution and bioactive delivery represent the most immediate application potential, yet challenges in process scale-up, shelf-life predictability, and regulatory compliance persist. Future efforts should therefore incorporate integrated in vivo/clinical studies, life-cycle assessments, and multiscale structure–function validation to bridge the gap between academic promise and industrial viability.

## 6 Conclusion and prospects

Pectin-based emulsion gels are soft-matter systems in which functional properties emerge from the coupled processes of emulsification and gelation. Preparation typically involves homogenization (HSSH, UH, HPH or MH) followed by acid-, ion- or enzyme-induced gelation of the continuous phase.Performance is evaluated through texture, bulk and interfacial rheology, microstructure, and stability under thermal, mechanical, freeze–thaw, gastrointestinal and long-term storage stresses. These measurements together link interfacial adsorption and network formation to macroscopic performance.

System behaviour depends on both pectin fine structure and the formulation environment. Molecular weight, degree and pattern of methoxylation, RG-I branching, and residual protein or feruloyl groups set the capacity for interfacial adsorption and network building; homogenization energy, oil-volume fraction, pH, ionic strength and protein co-solutes determine how much of that capacity is expressed. Classical, oil-free hydrogel models—centred on DM and simple acid or egg-box gelation—only partly explain emulsion gels. Above moderate oil fractions, droplets sterically disrupt long-range ionic bridges, while RG-I side chains can serve as interfacial anchors and entanglement sites—roles that HG-centric schemes largely overlook. Most studies still change one variable at a time in simplified systems, so transferable structure–interface–gel relationships under realistic food conditions remain limited.

  Several concrete research priorities follow from these limitations. First, quantitative structure–interface maps are still needed. Pectin structural features—DM, blockiness, RG-I side-chain length and branching, and residual protein or feruloyl content—should be systematically linked to interfacial strength, adsorbed-layer thickness and coalescence stability under controlled pH, ionic strength and oil fraction. Second, competing gelation forces—hydrogen bonding, hydrophobic association, Ca^2+^ bridging and covalent cross-linking—must be quantified in situ when acid, ions, oil and protein coexist, rather than treated as sequential, isolated regimes. Third, chemical tools should be applied more deliberately. Hydrophobic anchors can be enriched or better exposed to strengthen interfacial adsorption; overly long neutral-sugar side chains can be trimmed enzymatically to a moderate length to balance rapid adsorption with steric stabilisation; block-wise demethylation can favor ordered Ca^2+^ junctions without local over-cross-linking; and laccase- or transglutaminase-mediated pectin–protein links can build dual networks with improved thermal and freeze–thaw robustness.

Progress will also depend on more comparable experimental data. Many papers report only final droplet size or bulk storage modulus, without showing how homogenization alters molecular weight and side-chain structure, or how strong the interfacial film is before gelation. Without these intermediate measurements, it is unclear whether stability arises from a stronger interface, a denser continuous-phase network, or both. Future studies should therefore report, for the same formulation: (i) molecular weight and neutral-sugar profile before and after homogenization; (ii) interfacial dilatational and shear moduli at the pH and ionic strength used for gelation; (iii) oil fraction and droplet-size distribution; and (iv) bulk gel strength, water-holding capacity and storage stability over a defined time and temperature. Only when these data are collected together can laboratories build transferable rules linking process energy, interfacial structure and gel performance. Molecular simulation is a valuable complement, but it cannot replace this basic, aligned experimental set.

Application studies have advanced, yet the supporting evidence is still uneven. Pectin-based emulsion gels have moved beyond model systems in several directions: as fat replacers that approximate the texture of animal fat while improving fatty-acid profile; as carriers that protect lipophilic bioactives and support controlled release under simulated digestion; as 3D-printing inks with tunable rheology and shape fidelity; and as components of edible coatings that aid moisture control and shelf-life extension. These advances are genuine, but important gaps remain. Many delivery studies still equate improved in-vitro bioaccessibility with bioavailability—an overstatement that should be avoided until animal pharmacokinetic and, where appropriate, human data are available. Product-level trials in real foods are still fewer than laboratory formulations, and long-term storage, flavour release and sensory acceptance under practical processing conditions are thinly documented. Strengthening this evidence is as necessary as further structural work if application claims are to stay credible.

Cost and scale-up will shape how far these systems move beyond the laboratory. Food-grade pectin is among the higher-priced hydrocolloids, yet partial fat replacement in meat and bakery products already offers a workable balance when the gel supports a clear reduced-fat or improved fatty-acid claim and reduces animal-fat use. Full replacement and bioactive-loaded systems place higher demands on ingredient and process; their scope can widen as pectin is recovered more widely from fruit-processing residues and extraction is optimized—routes that techno-economic studies of by-product pectin already link to improved returns and shorter payback. Open cost–performance comparisons that link pectin dose and process intensity to shelf life and product value would give manufacturers a clearer basis for scale-up and steer laboratory design toward formulations that are both effective and economically realistic.

In summary, this review has examined the preparation, interfacial and gelation mechanisms, multiscale characterization, structural determinants and applications of pectin-based emulsion gels from a colloid and interface science perspective. By pairing current achievements with specific, actionable priorities, it aims to support more predictive design and a clearer path from laboratory formulation to practical use.

## CRediT authorship contribution statement

**Tingting Qi:** Writing – original draft, Resources, Conceptualization. **Jingnan Ren:** Writing – review & editing, Methodology, Formal analysis. **Siyi Pan:** Validation, Investigation. **Gang Fan:** Supervision, Project administration, Funding acquisition.

## Declaration of competing interest

The authors declare that they have no known competing financial interests or personal relationships that could have appeared to influence the work reported in this paper.

## Data Availability

All data supporting this systematic review were extracted from previous publications and are available within this review.
